# Genetics of Meesmann corneal dystrophy: a novel mutation in the keratin 3 gene in an asymptomatic family suggests genotype-phenotype correlation

**Published:** 2008-09-15

**Authors:** Jacek P. Szaflik, Monika Ołdak, Radosław B. Maksym, Anna Kamińska, Agnieszka Pollak, Monika Udziela, Rafał Płoski, Jerzy Szaflik

**Affiliations:** 1 Department of Ophthalmology, Medical University of Warsaw, Warsaw, Poland; 2 Department of Histology and Embryology, Medical University of Warsaw, Warsaw, Poland; 3 Institute of Physiology and Pathology of Hearing, Warsaw, Poland; 4 Department of Medical Genetics, Medical University of Warsaw, Warsaw, Poland

## Abstract

**Purpose:**

Juvenile epithelial corneal dystrophy of Meesmann (MCD, OMIM 122100) is a dominantly inherited disorder characterized by fragility of the anterior corneal epithelium and intraepithelial microcyst formation. Although the disease is generally mild and affected individuals are often asymptomatic, some suffer from recurrent erosions leading to lacrimation, photophobia, and deterioration in visual acuity. MCD is caused by mutations in keratin 3 (*KRT3*) or keratin 12 (*KRT12*) genes, which encode cornea-specific cytoskeletal proteins. Seventeen mutations in *KRT12* and two in *KRT3* have been described so far. The purpose of this study was to investigate the genetic background of MCD in a Polish family.

**Methods:**

We report on a three-generation family with MCD. Epithelial lesions characteristic for MCD were visualized with slit-lamp examination and confirmed by in vivo confocal microscopy. Using genomic DNA as a template, all coding regions of *KRT3* and *KRT12* were amplified and sequenced. Presence of the mutation was verified with restriction endonuclease digestion.

**Results:**

In the proband, direct sequencing of the polymerase chain reaction (PCR) product from amplified coding regions of *KRT3* and *KRT12* revealed a novel 1493A>T heterozygous missense mutation in exon 7 of *KRT3*, which predicts the substitution of glutamic acid for valine at codon 498 (E498V). Using PCR-Restriction Fragment Length Polymorphism (RFLP) analysis, the mutation was demonstrated to segregate with the disease (four affected members, three non-affected) and to be absent in 100 controls from the Polish population, indicating that it is not a common polymorphism.

**Conclusions:**

Location of the E498V mutation emphasizes the functional relevance of the highly conserved boundary motifs at the COOH-terminus of the α-helical rod domain in keratin 3 (K3).

## Introduction

Keratins are intermediate filament proteins that form a dense fibrous scaffold within the cytoplasm of epithelial cells. Based on the amino acid sequence, they are classified into type I or type II intermediate filaments, which are expressed in pairs and form obligate heterodimers in a tissue-specific and differentiation-specific manner. The predominant function of these structurally resilient polymeric proteins is to impart mechanical strength to the cells [1]. In addition, accumulating evidence suggests that keratins also have regulatory functions influencing cell size, proliferation, translation control, organelle transport, malignant transformation, and stress responses [2].

Mutations in keratin genes result in an abnormal fragility of epithelial cells, leading to their detachment, blistering of tissues in response to even mild physical trauma, and impaired keratinization [1,3]. Keratin mutations were detected in several human diseases affecting the epidermis and/or its appendages, e.g., epidermolysis bullosa simplex (a group of heritable skin blistering disorders), keratoderma disorders, and hair and nail defects. They were also found in extracutaneous epithelia such as mucosa and corneal epithelium [1].

The only known disorder associated with mutation in cornea-specific keratin 3 (*KRT3*) and keratin 12 (*KRT12*) representing type II and I intermediate filaments, respectively, is Meesmann corneal dystrophy (MCD) [4]. As with many other keratin disorders, MCD is inherited as an autosomal dominant trait with variable expression. The majority of mutations were found in *KRT12* and only two in *KRT3* [4-6]. All of them are located in the central α-helical rod domain responsible for protein heterodimerization and higher order polymerization. They cluster in the highly conserved boundary segments of the rod domain either within its NH_2_- (1A subdomain) or COOH- (2B subdomain) terminus [7].

MCD is characterized by fragility of the anterior corneal epithelium, which may lead to its recurrent erosions. Morphologically, the epithelium is disorganized and thickened with widespread cytoplasmic vacuolation and numerous small, round, keratin aggregate-laden intraepithelial microcysts [5,8]. They appear in childhood and increase in number with age. Although the disease is generally mild, some patients present with symptoms of lacrimation, photophobia, and intermittent diminution of visual acuity [8].

Here, we present the results of a clinical and molecular study of a previously unreported Polish family with MCD in whom a novel missense mutation in exon 7 of *KRT3* was found to segregate with disease. The E498V mutation affects a highly conserved amino acid [9] at the COOH-terminus of the K3 rod-domain and represents the third mutation to be detected in this gene. Other mutations found in *KRT3* and *KRT12* to date are also reviewed.

## Methods

A three-generation Polish family with four affected individuals was studied. All subjects gave informed consent in accordance with the tenets of the Declaration of Helsinki. A complete ophthalmological check-up including slit-lamp examination and confocal microscopy in vivo by Rostock Cornea Module (RCM) for HRT II (Heidelberg Engineering, Dossenheim, Germany), a preferred laser scanning confocal microscope for corneal epithelium evaluation [10], were performed.

Genomic DNA was extracted from blood obtained from all available family members (n=7). Control DNA samples came from a repository of anonymous samples (n=100, female:male ratio 1:1) representative of the background population of Central Poland, which has been described previously [11]. Ophthalmologic status of these individuals was not known.

DNA mutation screening was performed by amplifying the entire coding region of *KRT3* and *KRT12* with primers located in the noncoding sequences and designed based on the reference sequences of the respective genes, NM_057088 and NM_000223. Polymerase chain reaction (PCR) was performed at 94 °C for 2 min followed by 35 cycles at 94 °C for 45 s, 57–63 °C for 90 s, 72 °C for 60 s, and 72 °C for 10 min. Primer sequences and annealing temperatures for each primer set are given in Table 1. PCR products were examined on 1% agarose gels and then sequenced using an ABI PRISM 377 DNA sequencer (Applied Biosystems, Foster City, CA) and BigDye Termiantion cycle sequencing kit v. 3.1 (Applied Biosystems).

**Table 1:** Primers for polymerase chain reaction amplification and sequencing of *KRT3* and *KRT12*, their annealing temperature (Ta), and expected amplicon size.

**Gene/** **exon**	**Forward primer sequence (5’→ 3’)**	**Reverse primer sequence (5’→ 3’)**	**Ta (°C)**	**Product size (bp)**
*KRT3*				
exon 1	TgCACAggTCTTCATTTCCCATCC	TCCTCAACCCTggATATCTTCCCA	61	887
exon 2	AgTgTTgCCTgATgTTgCTTCCTg	ACCATgCTTggAgAAggAAggTgA	61	439
exon 3	ATggAgggAgggAAgAgATgAACT	ATTgCTCCAAAggCCTGAACTTgg	61	275
exon 4	gCTCTTTCTTgCTgCAgTTgTggT	gCACCAgCCTCAAATCTggAAACA	60	238
exon 5	AgTgAACAAgCTCCCTCTgTgTTg	TgAAACCTCCAgTggATCCCgTAA	60	235
exon 6	AAggTTTggTgggTgATgTTggAg	ATTTgTggAgATACTgCCCTgTgg	61	345
exon 7	AATCCATTgCATgTCAggAAgggC	TATCTGGCCCTTGGCCTATGACTT	60	354
exon 8	TgTTggTgATgTgCTTTgTgACgg	AAgCCAATCACTTCCCTCTCCTCT	60	228
exon 9	ACAATAACATAgCAgCTggCCTgg	AATACTCAgAggCCCggAgTgAAA	61	756
*KRT12*				
exon 1a	AgTgAACTTTTCAACTgCgA	TgCCCgAgAgAATACCTAgA	61.5	450
exon 1b	AggACTgggTgCTggTTAT	CTgCAAgTACAgCTAAATTggA	62	447
exon 2	TAgggCTTCAATCTTgTgTgTgTCCC	TTTATATCAATgAAggCAggACAgTAggAC	61.2	200
exon 3	CCCTCAACTgCTTTgCACTTggTT	CTCCATACTTgTCCTgACTCCAgA	58.4	289
exon 4	CAgggCCCACgAAAgTCACAAT	gTTCgCAggCCTTTCTgTgAATgT	58.3	272
exon 5	ACATTCACAgAAAggCCTgCgAAC	TggAAgTCCAAAggATgCTACgTC	58.4	235
exon 6	gCTCgTgCgCAAACAgACgT	CCCAggCATATCTTTACTAgA	60	490
exon 7	AgCCACCTgAACCACCTACTCTAA	AgCTATgAggTTACAggCATgAgC	63	469
exon 8	gCCTACATTAAACAACCAgTgTTgg	CAAgCAATCATCTTgCCTCTCAgC	61	684

The mutation in exon 7 was verified by polymerase chain reaction-restriction fragment length polymorphism (PCR-RFLP) analysis using HphI (MBI Fermentas, Vilnius, Lithuania). The PCR reaction was performed with the same primers as used for sequencing (Table 1), and the digestion was performed according to the manufacturer’s instructions. The digestion products were separated by electrophoresis in 2% agarose gels and visualized by ethidium bromide staining. In the presence of the E498V mutation, the digestion resulted in the bands of 304 bp and 260 bp whereas in the wild type homozygotes, only a 304 bp band was observed. The PCR-RFLP analysis was used to screen family members and 100 control DNA samples. All family members in whom the E498V mutation was found by PCR-RFLP were also analyzed by direct sequencing.

## Results

**Figure 1:**
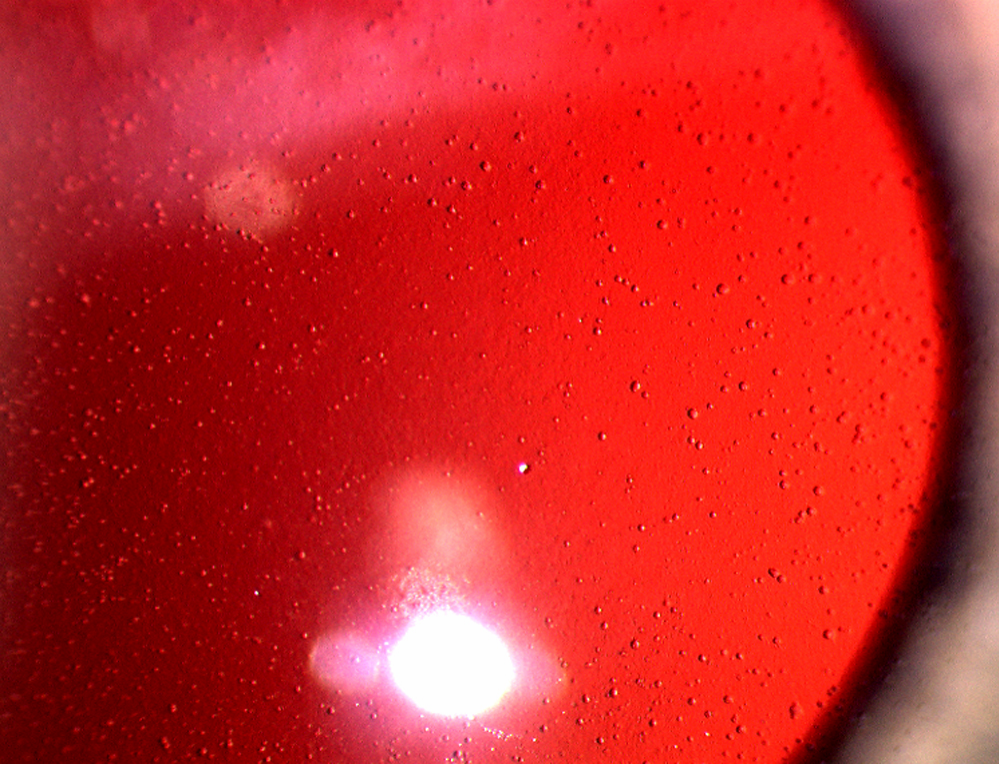
Slit-lamp photography of proband (III-1). This image demonstrates the microcystic appearance of the corneal epithelium.

**Figure 2:**
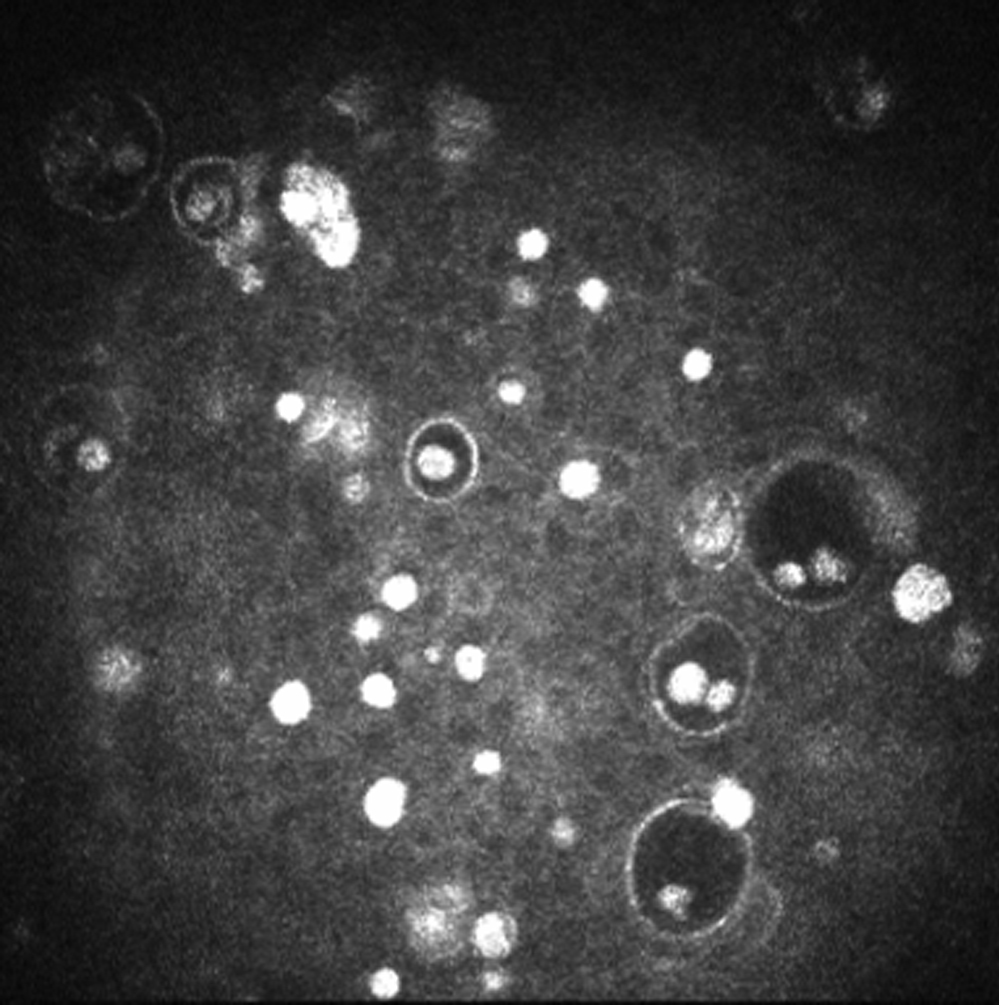
Confocal microscopy image of proband’s cornea. This image shows the presence of hyperreflective material within the intraepithelial cysts.

**Figure 3:**
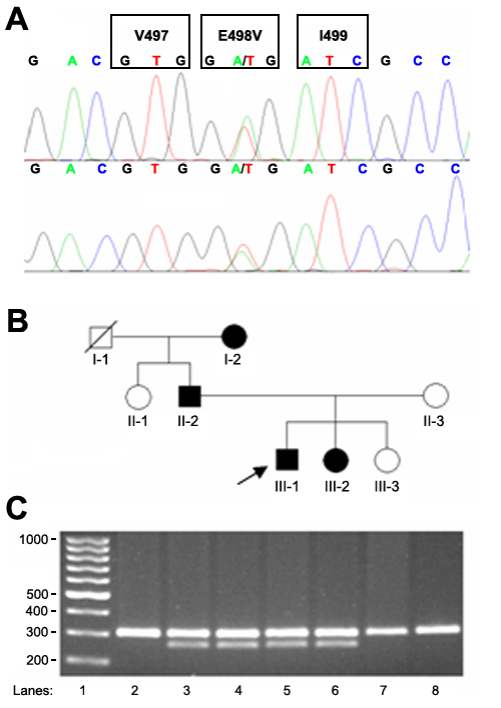
Identification of the heterozygous point mutation E498V in exon 7 of *KRT3* in the Polish MCD family. **A**: DNA sequencing. Electropherograms from bidirectional sequencing of *KRT3* exon 7 in the proband showed a 1493 A>T (GAG>GTG) heterozygous mutation, predicting the amino aid change E498V. **B**: Pedigree of the studied family. The arrow indicates the proband (III-1). **C**: PCR-RFLP analysis. The *KRT3* E498V mutation creates a recognition site for HphI. The presence of this restriction site is seen to cosegregate with MCD in this family. Upon digestion, the full sized 304 bp product is cut into bands of 260 bp and 44 bp (the latter not visible on the figure). DNA molecular weight markers are shown on the left (lane 1). The heterozygous E498V mutation (lanes 3–6) was detected in all affected family members (I-2, II-2, III-1, and III-2). The homozygous normal allele, represented by the 304 bp band (lane 2, 7, and 8), was found in unaffected family members (II-1, II-3, and III-3).

In the proband (III-1, 24 years old), the diagnosis of MCD was made on the basis of the typical clinical appearance of the corneal microcysts, which were detected in both eyes during routine eye examination (Figure 1 and Figure 2). They were also found in three other family members I-2 (71 years old), II-2 (49 years old), and III-2 (23 years old; Figure 3B). Patients I-2, II-2, and III-1 were hyperopic from early childhood. None of the affected family members complained about typical symptoms of MCD.

The pedigree of the examined family was consistent with an autosomal dominant mode of inheritance (Figure 3B). Since most mutations in MCD patients were found in exons 1 and 6 of *KRT12* and the remaining two in exon 7 of *KRT3*, these coding regions were initially sequenced in both the forward and the backward directions in the proband. The analysis revealed the presence of a novel heterozygous 1493A>T transversion in exon 7 of *KRT3* (Figure 3A). The mutation predicts a glutamate to valine amino acid change at codon 498 (E498V) within the region of highly conserved 2B rod domain segment in K3. Sequencing of the remaining coding regions of *KRT3* in the proband did not show any alterations.

The E498V mutation creates a recognition site for the HphI endonuclease. The PCR-RFLP analysis showed that the E498V mutation cosegregated with the MCD phenotype in the studied family members (Figure 3B,C). Using this method, DNA samples from 100 subjects from the background population of central Poland were also screened, and no carriers were found.

Sequencing of *KRT12* exon 1 of from the proband revealed the presence of a common coding homozygous non-synonymous single nucleotide polymorphism (rs11650915). Additionally, a previously unreported heterozygous substitution of A>C in the 3′ region of *KRT12* (position NT:2741849, chromosome 17:36271079, according to Genewindow) was detected. The latter variant did not segregate with the disease. It was found in affected members (III-1 and III-2) as well as unaffected family members (II-3 and III-3).

## Discussion

The E498V mutation in *KRT3* found in the Polish family with MCD affects a strongly conserved residue within the 2B subdomain of the intermediate filament chain [9]. Conservation of an amino acid residue indicates its significance for protein function and low tolerance to replacement [9,12]. The E498V mutation predicts a particularly unfavorable substitution of a negatively charged, polar glutamate to an aliphatic and hydrophobic valine. Glutamate is often present in the protein active or binding site. It pairs with positively charged amino acids to create hydrogen bonds, which are important for protein stability. Conversely, valine is preferably present in protein hydrophobic cores. It contains two substituents at its C-beta carbon, which restrict the conformational changes that the main chain can adopt. One of the most pronounced effects of this property is the difficulty of valine to adopt an α-helical conformation [12]. Thus, the replacement of glutamate to valine is likely to influence both the physicochemical and structural properties of the α-helical rod domain in K3, leading to the disruption of the cytoskeletal keratin network.

**Figure 4:**
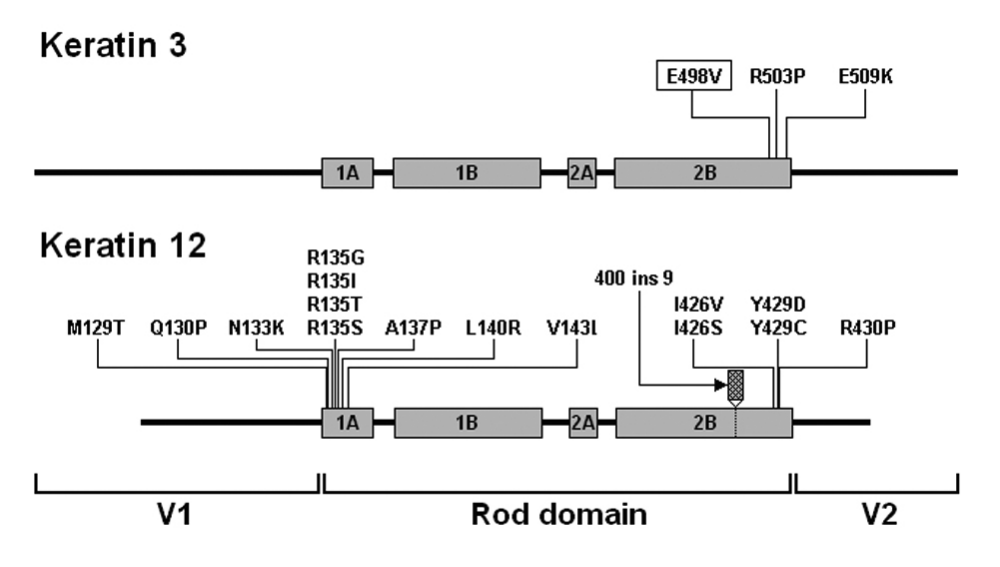
Schematic drawing of K3 and K12 structure with assigned positions of the published mutations. Keratins are composed of three main parts, the central α-helical rod domain, which is divided into four subdomains (1A, 1B, 2A, and 2B), and the two non-helical variable domains (V1 and V2) at each end [3]. All three mutations within *KRT3* localize exclusively in the boundary motif of the 2B subdomain. Among the mutations in *KRT12,* 11 were found in the 1A subdomain and six in the 2B subdomain (see also Table 2).

All mutations in *KRT3* and *KRT12* reported to date affect one or the other terminus of the central α–helical rod domain and all but one (a 27 bp insertion [13]) are missense mutations (Table 2, Figure 4). Of note is also the lack of reported mutations in the 1A subdomain of K3. Whether this is only a chance finding resulting from the scarcity of genotyped MCD cases or their incompatibility with normal development, which seems less plausible, remains to be elucidated.

**Table 2:** *KRT3* and *KRT12* genotypes and symptoms in patients with MCD.

**Gene/exon**	**Nucleotide**	**Protein**	**Ocular symptoms**	**Reference**	
*KRT3*					
exon 7	1493A>T	E498V	asymptomatic	present study	
exon 7	1508G>C	R503P	foreign body sensation, mild blurred vision	[6]	
exon 7	1525G>A	E509K	-	[4]	
*KRT12*					
exon 1	410T>C	M129T	-	[14,15]	
exon 1	413A>C	Q130P	recurrent painful erosions, foreign body sensation, photophobia, lacrimation, blurred vision	[16]	
exon 1	423A>G	N133K	soreness of both eyes; deterioration in visual acuity	[17]	
exon 1	427A>G	R135G	photophobia, lacrimation, itching	[18]	
exon 1	428G>T	R135I	photophobia, lacrimation, itching	[18]	
exon 1	428G>C	R135T	-	[4,14]	
exon 1	429A>C	R135S	post-traumatic recurrent erosion	[13]	
exon 1	433G>C	A137P	photophobia	[19]	
exon 1	443T>G	L140R	photophobia, lacrimation, itching	[18]	
exon 1	451G>C	V143L	-	[4]	
exon 1	451G>T	V143L	asymptomatic	[20]	
exon 6	1222+ATCAGCAACCTGGAGGCACAGCTGCTC	400 ins ISNLEAQLL	recurrent erosions, foreign body sensation, photophobia, fluctuating vision, contact lens intolerance		[13]
exon 6	1300A>G	I426V	asymptomatic		[21]
exon 6	1301T>G	I426S	photophobia		[15]
exon 6	1286A>C	Y429D	photophobia, lacrimation, itching		[18]
exon 6	1286A>G	Y429C	recurrent erosions, foreign body sensation, photophobia, lacrimation, fluctuation of visual acuity		[6]
exon 6	1289G>C	R430P	symptoms from birth; photophobia, lacrimation, periodic burning, irritation, significant impairment of visual acuity		[7]

An interesting and yet unresolved issue in MCD is the occurrence of asymptomatic cases despite the presence of proven *KRT* mutations and morphological findings. An example of such MCD presentation is the family reported in this study. Interestingly, our case and the review of data on the mutations and phenotypes reported so far in MCD (Table 2) and other diseases caused by keratin mutations may suggest a framework for understanding genotype/phenotype correlation in MCD.

Amino acids located in the boundary sequence motifs of the keratin rod domain are highly conserved and particularly important in intermediate filaments assembly as they mediate end-to-end interactions between keratin heterodimers and filament elongation. These regions were found to represent mutational hot spots in MCD as well as in other keratin types [3]. mutations, which affect the boundary sequence motifs,  typically exert a dominant-negative effect being highly disruptive to filament assembly and usually associate with the severe disease phenotypes. In contrast, mutations in other parts of keratin genes are compatible with filament assembly, and the disease phenotype is generally milder [5]. Interestingly, all three mutations, which so far have been reported in asymptomatic patients (*KRT12*; V143L, *KRT12*; I426V, and *KRT3*; E498V) or patients with relatively mild symptoms (*KRT12*; I426S), are located innermost relative to other mutations and possibly in less critical regions of the boundary motifs of their respective subdomains (Figure 4). The only exception is the *KRT12* 400ins9 mutation, which is not directly comparable to other mutations since it leads to an insertion of nine novel amino acids and is likely to be damaging to filament assembly despite a relatively long distance from the terminus of the 2B subdomain (Figure 4). These data suggest that putative missense mutations localized internally in *KRT12* (V143 and I426) or *KRT3* (E498) are also likely to be asymptomatic and thus provide a general framework for genotype/phenotype correlation in MCD.
